# Ultrasonic synthesis of nano-PrO_1.8_ as nanozyme for colorimetric determination of *trans*-resveratrol

**DOI:** 10.1038/s41598-020-61452-x

**Published:** 2020-03-10

**Authors:** Lizhi Wang, Yang Liu, Chanfang Lu, Zhouping Yang, Yaqing Liu, Yanying Wang, Hanbing Rao, Wei Zhang, Xianxiang Wang

**Affiliations:** 1 Institute of Animal Nutrition, Sichuan Agricultural University, Chengdu, 611130 Sichuan China; 2 College of Science, Sichuan Agricultural University, Chengdu, 611130 Sichuan China; 3 College of Veterinary Medicine, Sichuan Agricultural University, Chengdu, 611130 Sichuan China

**Keywords:** Biosensors, Biosensors, Sensors, Sensors

## Abstract

In this study, nano-PrO_1.8_ were synthesized successfully in ionic liquids (ILs) as template assisted ultrasonic irradiation method. Various precipitating agents and different types of ILs were investigated to determine their respective effects on the morphology of the end products. Using hydrazine hydrate as a precipitating agent and 1-carboxymethyl-3-methylimidazolium chloride as a template, spherical structure with an average diameter of 250 nm was obtained. It is worth noting that the prepared material exhibits high peroxidase-like activity and weak oxidase activity. Then, the catalytic oxidation capacity of the nano-PrO_1.8_ was evaluated by the peroxidase substrate 3,3′,5,5′-tetramethylbenzidine (TMB). The colorless of TMB can be converted into blue oxidized TMB (oxTMB) in the presence of nano-PrO_1.8_, but *trans*-resveratrol inhibited its peroxidase-like activity and weakened the blue color. Hence, we developed a sensitive, selective and simple colorimetric method for *trans*-resveratrol detection using nano-PrO_1.8_ as peroxidase-like enzyme. A linear relationship was found in the range of 0.30 µM–16 µM *trans*-resveratrol with the detection limit of 0.29 µM. Satisfactory results were achieved when the method was submitted to the determination of *trans*-resveratrol in white wine samples.

## Introduction

Nanozymes have many advantages over natural enzymes, such as their high stability under harsh environmental conditions, bulk-scale and low-cost synthesis. Therefore, nanozymes have been used for broad applications, including biomolecular detection^1,2^, disease treatment^3,4^, environmental protection^5^ and antibacterial agents^6^. In the past decades, a lot of nanomaterials, including ferromagnetic nanoparticles, fullerene derivatives, gold nanoparticles or clusters, and rare earth nanoparticles, have been found to exhibit unexpected enzyme-like activity^7^. Among them, ferromagnetic nanomaterials are the earliest developed nanozymes with good peroxidase properties, and are widely used in colorimetric analysis^8,9^. Numerous efforts have been devoted to researching main elements, but actinide elements have received relatively little attention. Looking for new nanozymes is still the goal pursued by chemists. To date, Solvothermal^10^, reduction^11^, microwave^12^ and other methods have been used for the synthesis of nanozymes. Compared with these synthetic methods, the ultrasound-assisted synthesis and sonochemistry method is efficient and alternative to conventional methods for the synthesis of nanomaterials, due to the acoustic cavitation caused by the sonochemical reaction in which the bubbles expand and collapse rapidly when sound waves pulse through a liquid. The cavitation process consists of the creation, growth, and implosive collapse of gas vacuoles in a solution^13^. In addition, the use of ionic liquids (ILs) as templates and reactants with the aid of ultrasound for fabricating inorganic materials is also quite interesting. ILs possess low toxicity, high ionic conductivity, high thermal stability, very low vapor pressure, and ability to dissolve a variety of materials, and they are being explored as green solvent to substitute conventional volatile organic solvents in a variety of processes^14,15^. ILs are able to act as a template in the manufacturing of nanomaterials because of their pre-organized structures^16^. For example, porous inorganic nanomaterials^17^ and mesoporous silica^18^ have been prepared successfully with ILs templates. Combining the convenience of sonochemical synthesis with the excellent properties of ILs, a simple, effective, mild, and surfactant-free method for morphology controllable synthesis of nanomaterials was developed in the past years^19^.

**Figure 1:**
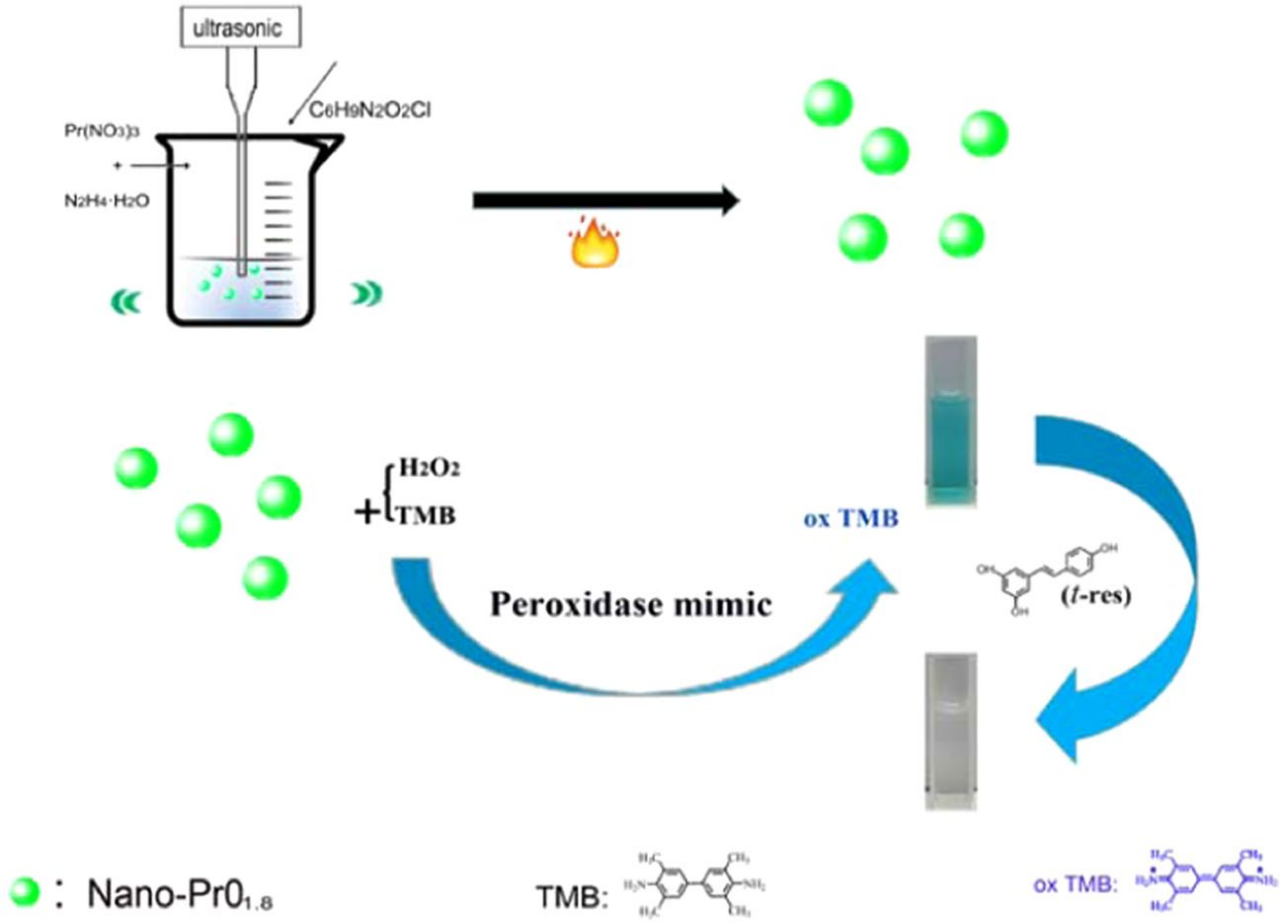
Overall synthesis procedures of the nano-PrO_1.8_ under ultrasonic field and their as peroxidase-like for *trans*-resveratrol colorimetric detection.

*Trans*-resveratrol, a polyphenol nanoflavonoid compound, has been discovered and well-documented in recent years to have potential therapeutic effects based on its bioactivity, such as anti-oxidant, anti-inflammatory, anti-aging, antitumor, and anti-mutagenic, etc^20^. Nowadays, people are more and more concern that products they eat or drink may have positive or negative effects on their heath. There is a general trend for the healthy life style in order to slow down the aging process. *Trans*-resveratrol as a star molecule, the level in food or wine is attracted people’s attention naturally. Therefore, it’s determination and quantification are of very important. Many detection methods are reported for determination of *trans*-resveratrol, including high-performance liquid chromatography (HPLC)^21^, capillary electrophoresis (CE)^22^, and gas chromatography (GC)^23^. Hence, developing a more simple and fast analysis method for *trans*-resveratrol is important for clinical analysis. In general, the colorimetric analysis methods have many merits including the simple and easy procedures, mild reaction conditions and low cost. Besides, the color changes can be visually observed by naked eye on-site. In the present paper, we reported a facile strategy to prepare nano-PrO_1.8_ by sonochemical synthesis in an ILs, and found the synthesized nano-PrO_1.8_ had a good peroxidase-like activity and weak oxidase-like activity. As a result, the developed PrO_1.8_ was able to catalyze the peroxidase substrate TMB into blue oxidized oxTMB, and *trans*-resveratrol inhibited this catalytic effect and weakened the blue color. Hence, a simple and sensitive colorimetric sensing platform was established to determine the concentrations of *trans*-resveratrol on the color change. Furthermore, the analytical method was also successfully employed in white wine sample (Fig. 1).

## Experiment Section

### Materials and instruments

All ILs were purchased from Kekaite Co. (Lanzhou, China). Reagent grade depleted Pr(NO_3_)_3_ and 3,3,5,5-tetramethylbenzidine (TMB) were obtained from Macklin Co. Ltd. (Shanghai, China). The *trans*-resveratrol was purchased from Solarbio Life Science (Beijing, China). All other chemicals, including hydrazine hydrate, ethylenediamine, H_2_O_2_ and ethanol, were of analytical grade and used without further purification.

Ultrasound synthesis was assisted by an ultrasonic homogenizer (220 V, 950 W) (Ningbo Scientz Biotechnology Co., Ltd.). Powder X-ray diffraction (XRD) analyses were performed on a Bruker D8 Advance diffractometer with Cu Kα radiation, and scanning electron microscopy (SEM) observations were performed with a Hitachi S-4800 scanning electron microscope, and the morphology and sizes of as-prepared materials were analyzed with JEOL 2100 high resolution transmission electron microscope (HRTEM) (JEOL, Japan). An EMGA-920 Oxygen Elemental Analyzer (HORIBA, Japan) was used to determine the oxygen content. The chemical composition of the products was examined with X-ray photoelectron spectrometer (XPS, ESCALAB 250Xi, ThermoFisher). UV-Visible absorption spectra were recorded on a UV-A390 spectrometer (AOE Instruments, Shanghai, China) with 1.0 cm path length.

### Synthesis of nano-PrO_1.8_ nanocrystal

In a typical synthesis, 0.20 g of Pr(NO_3_)_3_ and 0.1624 g of 1-carboxymethyl-3-methylimidazolium chloride ([HO_2_CMMIm]Cl) were dissolved in 40 mL water. Then, 500 μL of N_2_H_4_·H_2_O was added with a magnetic stirrer, and a slurry-like green suspension was formed in the glass beaker. Then, the glass beaker was placed in an ultrasonic homogenizer at 45% power (427.5 W) for 30 minutes, and next the beaker was cooled naturally to room temperature until a dark green powder was formed at the bottom of the beaker. The powder was obtained after the green powder was centrifuged, filtered, rinsed several times with water and ethanol, and calcinated at 800 °C for 4 hours in a muffle furnace finally. The control experiments were performed by adjusting the types of ILs ([HOOCMMIm]Cl and [HMIm]I) and precipitating agents (C_2_H_8_N_2_ and N_2_H_4_.H_2_O).

### Peroxidase-like activity and enzyme kinetic analysis of nano-PrO_1.8_

The peroxidase-like activity of the nano-PrO_1.8_ was performed at varied temperatures and pH using 1 mg/mL PrO_1.8_ with 4 mM TMB and 100 mM H_2_O_2_ as substrate. Ultrapure water was used in this experiment to avoid the influence of other ions on the activity of the nano-PrO_1.8_, and the pH of the reaction system was adjusted with a 0.2 M sodium acetate buffer solution. Then the enzyme kinetic analysis of the reaction was carried out by recording the absorption spectra in 652 nm at time scan mode. Unless otherwise indicated, the reaction was carried out at 40 °C in 2.55 mL acetic acid buffer solution (0.2 M, pH 3.5) using 1 mg/mL nano-PrO_1.8_. For the TMB kinetic assays, 100 mM H_2_O_2_ was added to a 2.55 mL buffer solution with varying TMB concentrations, and for the H_2_O_2_ kinetic assays, TMB (5 mM) was added to a 2.55 mL buffer with varying H_2_O_2_ concentrations. The kinetic parameters were calculated based on the Michaelis-Menten equation: ${\rm{V}}=\frac{{{\rm{V}}}_{{\rm{\max }}}\times [S]}{{K}_{{\rm{m}}}+[S]}$.

Where *V* is the initial velocity, V_max_ is the maximal reaction velocity, [*S*] is the substrate concentration and *Km* is a Michaelis constant.

To study the influence of reaction buffer pH on the catalytic activity of nano-PrO_1.8_, the pH of 0.1 M acetic acid buffer solution varying from pH 2.6 to 7.9 was investigated at 40 °C. To compare the influence of temperature on the nano-PrO_1.8_ catalytic activity, the catalytic reactions were incubated in different temperature water bath from 25 to 80 °C under pH 3.5. The relative activity was defined as: Relative activity (%) = A_1_/A_2_ × 100, where A_2_ was the maximum absorbance, and A_1_ was the sample absorbance measured at the same conditions.

### Colorimetric detection of *trans*-resveratrol

Under the optimum conditions, 150 μL of nano-PrO_1.8_ (dispersed with 30% ethanol, the concentration was 1 mg/mL), 3.33 mM H_2_O_2_ and 1.03 mM TMB were added in 0.2 M acetic acid buffer (2.55 mL, pH = 3.5) solution. Then, a *trans*-resveratrol was added in different concentrations (final concentration of *trans*-resveratrol was 0.292 μM to 16.06 μM). All the test groups were mixed and incubated at 40 °C for 15 minutes, then the absorbance was determined by UV-Vis spectrophotometer at 652 nm. The acetic acid buffer solution was used instead of *trans*-resveratrol solution as the blank sample.

## Results and Discussion

### Characterization of nano-PrO_1.8_

**Figure 2:**
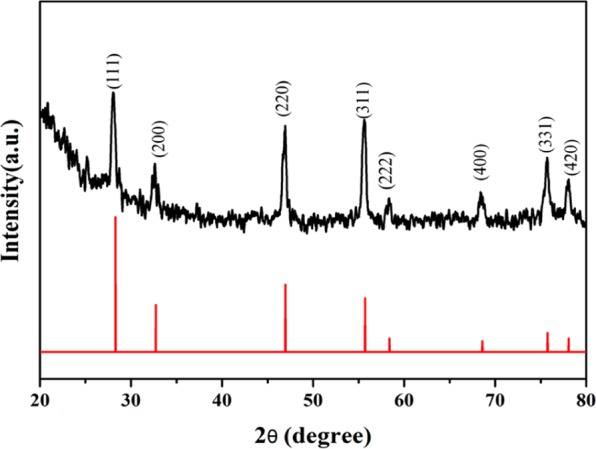
XRD pattern of nano-PrO_1.8_.

The nano-PrO_1.8_ particles were characterized by XRD, SEM, HRTEM, XPS and Oxygen Elemental Analyzer. XRD patterns was displayed elemental fractions of nano-PrO_1.8_ of Fig. 2. The prepared material has the main peaks at 28.8°, 32.7°, 46.9°, 55.7°, 58.4°, 68.6°, 75.8° and 78.1°, respectively relating to the (111), (200), (220), (311), (222), (400), (331), and (420) planes. This observation is in good agreement with the standard JCPDS 06-0639. To further confirm the oxygen content in praseodymium oxide, the prepared powder was analyzed by EMGA-920 Oxygen Elemental Analyzer. The sample in an inert atmosphere was heated to gas, which passes by the carbon dioxide converter where oxygen reacts with carbon, forming CO_2_ and is measured by infrared absorption. The result of oxygen concentration was 16.95 ± 0.43%, which proved the oxidation state of praseodymium is PrO_1.8_.

**Figure 3:**
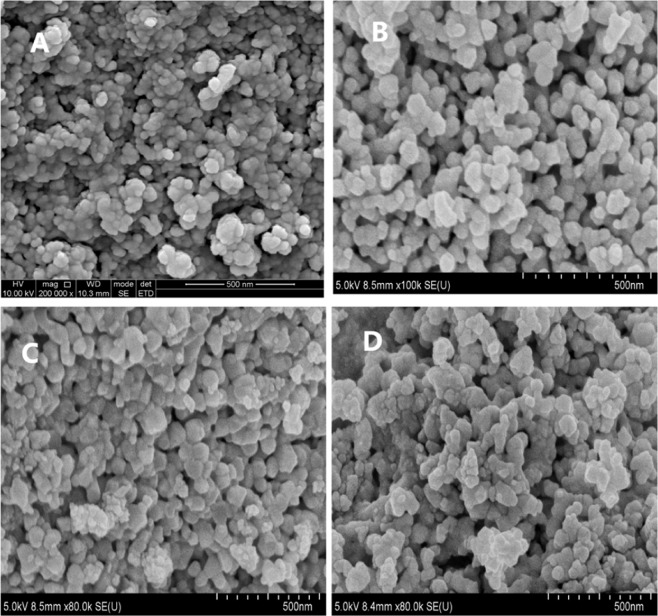
SEM images of products synthesized with different precipitating agents and ILs. (**A**) N_2_H_4_.H_2_O and [HOOCMMIm]Cl, (**B**) NaOH and [HOOCMMIm]Cl, (**C**) C_2_H_8_N_2_ and [HOOCMMIm]Cl (**D**)NaOH and [HMIm]I.

**Figure 4:**
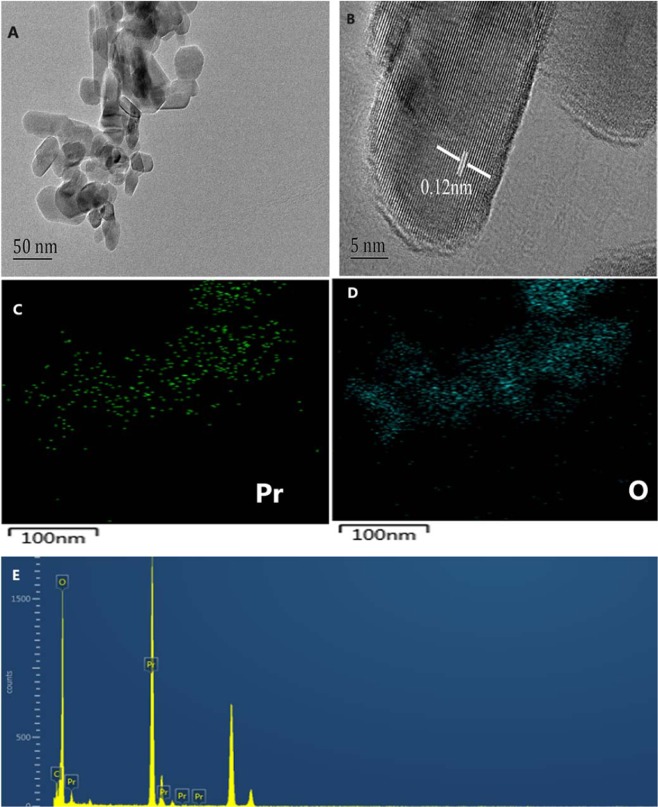
HRTEM (**A,B**) and mapping images (**C,D**) and (**E**) EDX spectrum of the PrO_1.8_ prepared from N_2_H_4_.H_2_O and [HOOCMMIm]Cl.

The impacts of the ILs types and precipitating agents on final products morphology were compared in the experiment. Three types of precipitating agents (N_2_H_4_.H_2_O, NaOH and C_2_H_8_N_2_) and two types of ILs ([HOOCMMIm]Cl and [HMIm]I) were used to study the effect on morphology of the products. The molar ratio of Pr(NO_3_)_3_ to the ILs was 1:2 and the calcination temperature was 800 °C for 4 hours. Figure 3 shows the SEM images of the products prepared with different precipitating agents and ILs. The results demonstrated that the morphology of the products was a well-crystallized spherical or hexagonal shape and had little dependence on the precipitating agents and ILs types. The morphology of the material prepared from N_2_H_4_.H_2_O and [HOOCMMIm]Cl was also characterized by HRTEM. As shown in Fig. 4A, the material has a hexahedral structure, and the lattice spacing of the material is 0.12 nm (Fig. 4B). In addition, the element distribution was analyzed by mapping images. The distribution of Pr and O elements in materials is uniform (Fig. 4C,D). The EDX (Energy Dispersive X-ray) spectrum also shows that the main element in the prepared material is Pr and O (Fig. 4E).

**Figure 5:**
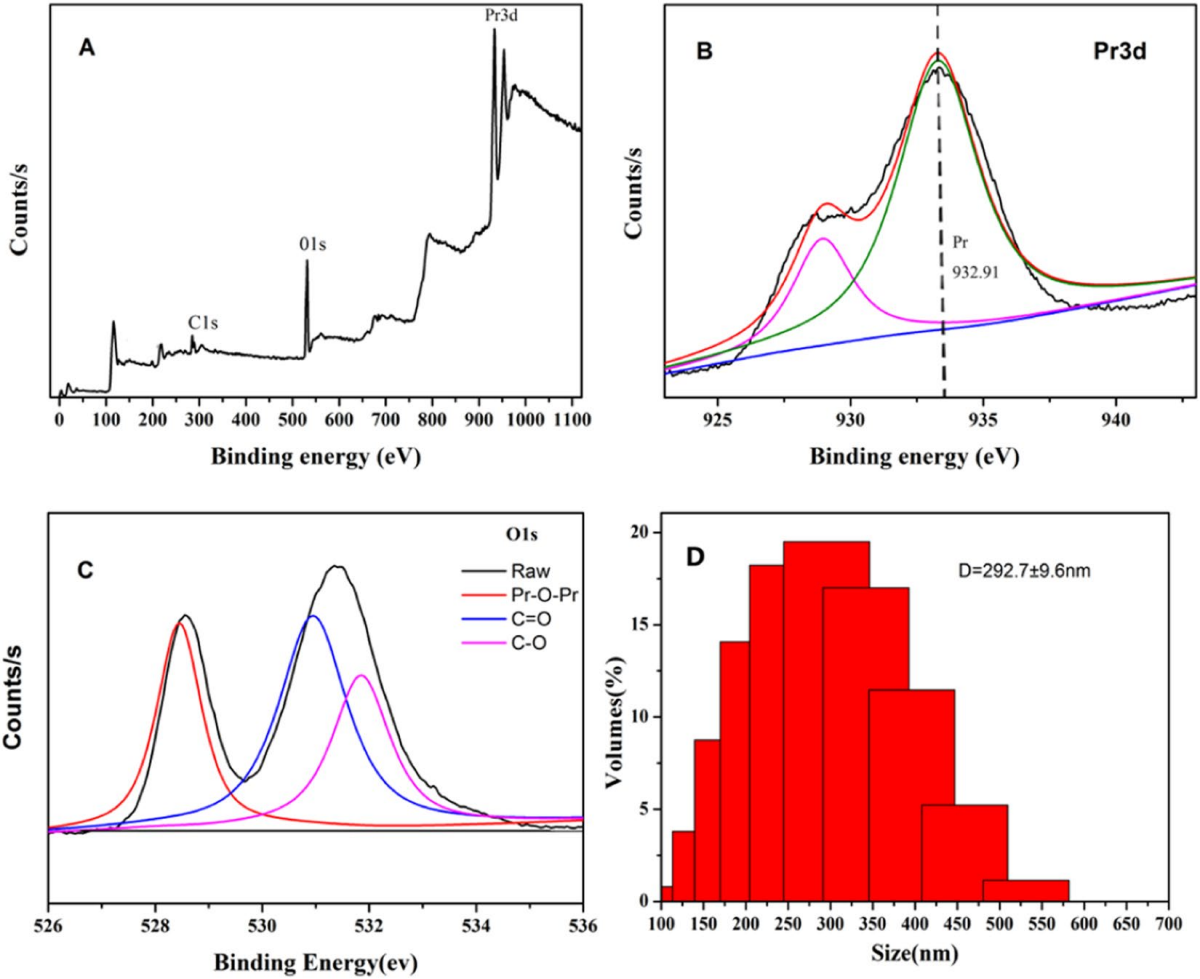
XPS analysis of nano**-**PrO_1.8_ (**A**) survey spectrum, (**B**) Pr 3d, (**C**) O1s and (**D**) DLS analysis of nano**-**PrO_1.8_.

In order to determine the valence of the various elements in nano-PrO_1.8_, XPS study was carried out. The survey spectra of nano-PrO_1.8_ confirmed that the presence of Pr and O. The binding energies (BEs) were calibrated using the C 1 s energy of 284.6 eV. The single C 1 s peak (Fig. 5A) is attributed to adventitious carbon that seems to exhibit an unavoidable presence on all air exposed materials. The binding energies for Pr3d at 932.91 eV (Fig. 5B), are in good agreement with the literature^24^. The O1s spectrum (Fig. 5C) is broad and asymmetric and can be deconvoluted into three peaks, which indicate the presence of three different oxygen species, there are attributed to C-O, C=O groups^25^ and Pr-O-Pr groups. The DLS (Dynamic Light Scattering) test results are shown in Fig. 5D. The results show that the particle size of the material is approximately normal distribution, the particle size of the material is between 100 and 550 nm, and the range of particle size distribution at 292.7 nm is the largest.

### Peroxidase-like activity and oxidase-like activity of the material

**Figure 6:**
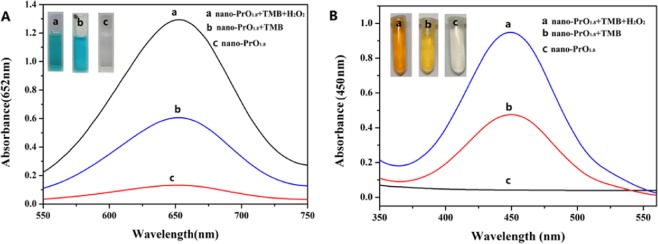
Typical absorption curve of different reaction systems using (**A**) TMB and (**B**) o-diaminobenzene as the reaction substrate.

The peroxidase-like and oxidase-like activity of nano-PrO_1.8_ were tested by oxidizing its enzyme substrate TMB and o-phenylenediamine in the presence and absence of H_2_O_2_ at room temperature, respectively. The experimental results show that when the precipitating agent is hydrazine hydrate, the product maintains the highest catalytic activity under the same experimental conditions. Figure 6 shows the typical absorption curves of the different reaction systems. The nano-PrO_1.8_ can catalyze the oxidation of colorless TMB in the absence of H_2_O_2_ to produce bright blue color, indicating that the nano-PrO_1.8_ has oxidase-like activity (Fig. 6A). However, when *trans*-resveratrol (*t-*res.) was added into the solution, the catalytic activity of the nano-PrO_1.8_ was rapidly lowered due to the suppression of oxidase-like activity, and the blue color was faded. Similar experimental results can also be observed in the presence of H_2_O_2_, indicating that the nano-PrO_1.8_ have peroxidase-like activity (Fig. 6A). Simultaneously, the prepared nano-PrO_1.8_ can catalyze the oxidation of o-diaminobenzene in the presence of H_2_O_2_ or in the absence of H_2_O_2_ (Fig. 6B). The absorbance value in the presence of H_2_O_2_ is higher than in the absence of H_2_O_2_ at the same conditions, this indicates that the activity of peroxidase-like is higher than oxidase-like under the same conditions. Thus, we only studied the peroxidase-like activity of nano-PrO_1.8_ and analytical application in the next experiments.

**Figure 7:**
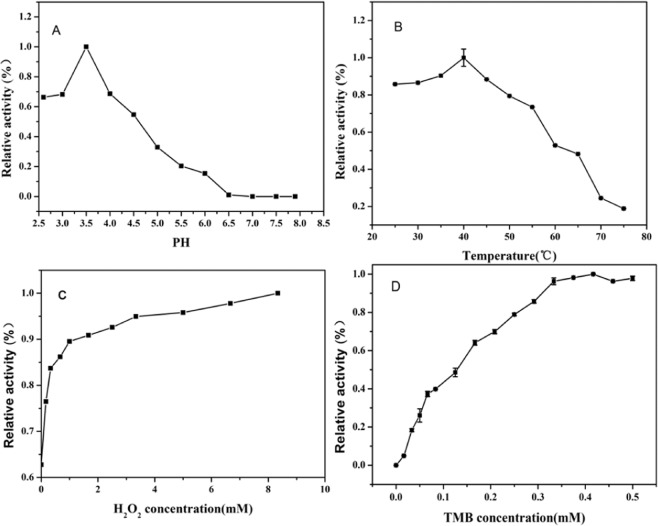
The catalytic activity of the nano-PrO_1.8_ with TMB as substrate were affected by (**A**) pH, (**B**) temperature, (**C**) H_2_O_2_ concentration and (**D**) TMB concentration.

Then, the peroxidase-like catalytic activity of the nano-PrO_1.8_ using TMB as the substrate was proved to be dependent on the pH values, temperature, concentrations of H_2_O_2_ and concentrations of TMB (Fig. 7). These results demonstrated that the nano-PrO_1.8_ have similar catalytic activities with nature enzyme, with maximum activity in a mildly acidic (pH at 3.5), negligible catalytic activity lost over a range of temperature (25–55 °C), and maximum activity at a 6.5 mM concentration of H_2_O_2_. Therefore, nano-PrO_1.8_ with peroxidase-like activity can be used in broad ranges of pH and temperature.

### The kinetic assay of nano-PrO_1.8_

**Figure 8:**
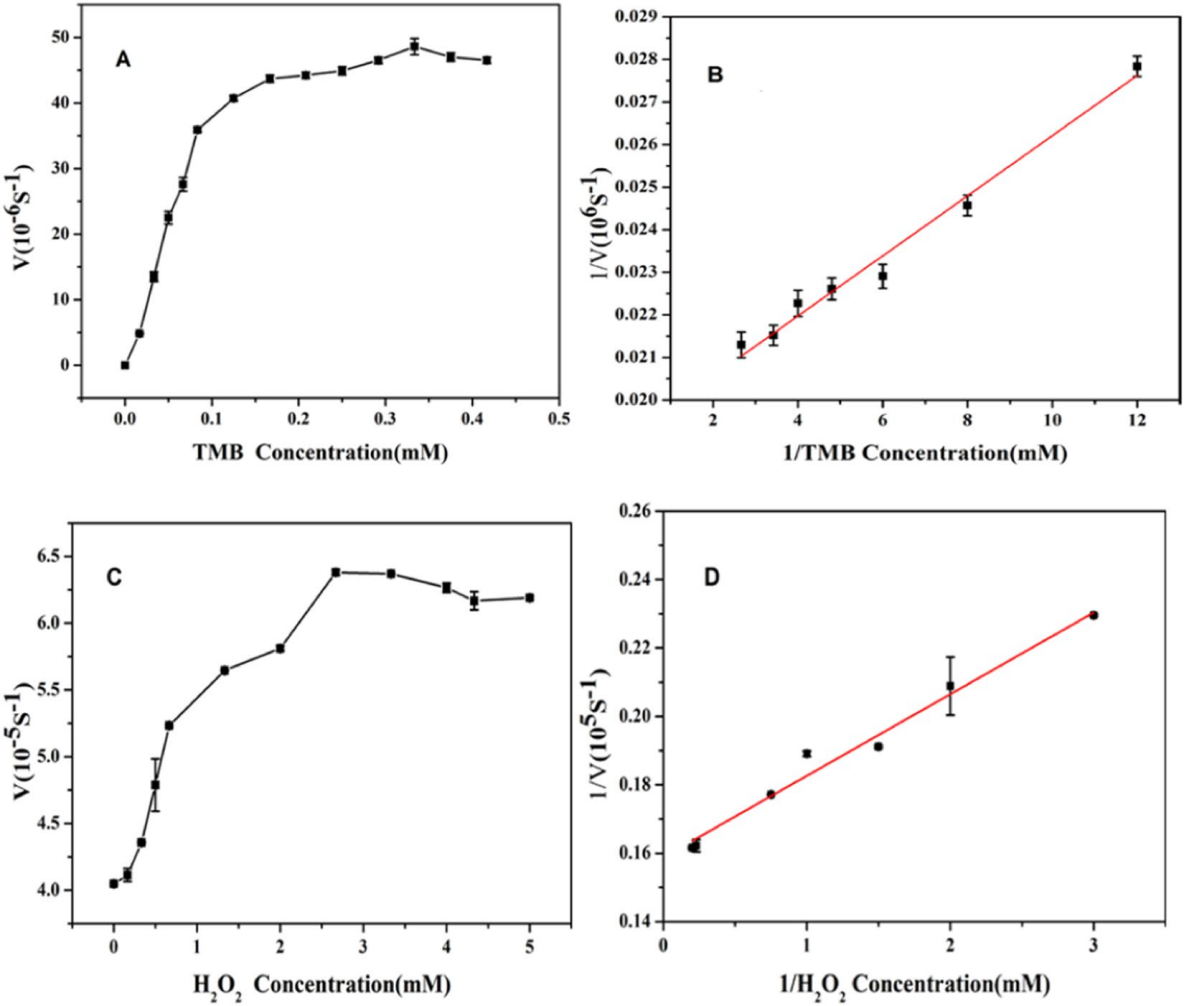
Steady-state kinetic assay of nano-PrO_1.8_. (**A**) changing the concentrations of TMB with 3.3 mM H_2_O_2_, (**B**) double-reciprocal model of the concentration of TMB, (**C**) changing the concentrations of H_2_O_2_ with 0.33 mM TMB and (**D**) double-reciprocal model of the concentration of H_2_O_2_.

In order to study the kinetics of the peroxidase-like catalytic activity of nano-PrO_1.8_, the catalytic oxidation of the oxidase substrate TMB was carried out in the presence of H_2_O_2_. Like some natural enzymes, the catalytic activity of nano-PrO_1.8_ was further investigated based on the enzyme kinetics theory, in which H_2_O_2_ and TMB were the substrates under the optimal conditions, as shown in Fig. 8. The evident steady-state kinetic parameters V_max_ and K_m_ by applying a reciprocal plot^26^: 1/v = (K_m_/V_max_) (1/[S]) + 1/V_max_, demonstrated in Table 1. The results show that the K_m_ of nano-PrO_1.8_ for TMB is 0.0367 mM, and the k_m_ of nano-PrO_1.8_ with H_2_O_2_ is 0.1502 mM, indicating that the Km of the nano-PrO_1.8_ with TMB was smaller than that of HRP and the nano-PrO_1.8_ possess better affinity for TMB than horseradish peroxidase (HRP) studied previously^8^. Furthermore, the Km of the nano-PrO_1.8_ with H_2_O_2_ is also smaller than that of the HRP, implying that nano-PrO_1.8_ possess higher affinity for H_2_O_2_ than HRP. Similarly, we also compared the K_m_ and V_max_ of other reported nanozyme. The results showed that the mimic enzyme activity of nano-PrO_1.8_ was stronger than these reported materials.

**Table 1:** Comparison of Km and Vmax of nanozyme and HRP.

Catalyst	Substrate	Km (mM)	V_max_ (M s^-1^)	Ref.
HRP	TMB	0.434	2.01 × 10^−8^	^8^
HRP	H_2_O_2_	3.7	3.34 × 10^−8^	^8^
Fe_3_O_4_ MNPS	TMB	0.098	3.44 × 10^−8^	^8^
Fe_3_O_4_ MNPS	H_2_O_2_	154	9.78 × 10^−8^	^8^
BSA-Au NCs	TMB	0.00253	6.23 × 10^−8^	^29^
BSA-Au NCs	H_2_O_2_	25.3	7.21 × 10^−8^	^29^
nano-PrO_1.8_	TMB	0.0367	0.521 × 10^−8^	This study
nano-PrO_1.8_	H_2_O_2_	0.1502	0.006298 × 10^−8^	This study

### *Trans*-resveratrol detection using TMB-H_2_O_2_-nano-PrO_1.8_ system

**Figure 9:**
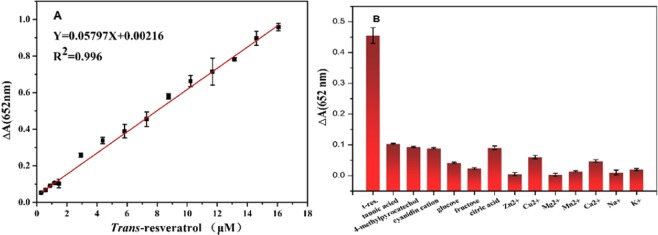
(**A)** The dose–response curves for UV-Vis detection of *trans*-resveratrol using nano-PrO_1.8_ as the peroxidase-like activities and (**B**) selectivity toward *trans*-resveratrol of the TMB-H_2_O_2_-nano-PrO_1.8_ system against potential interferences.

Based on the previous experimental results that *trans*-resveratrol can effectively inhibit the activity of peroxidase-like of nano-PrO_1.8_, *trans*-resveratrol was detected in white wine with TMB-H_2_O_2_-nano-PrO_1.8_ system. The results showed a linear relationship between *trans*-resveratrol concentration and ΔA, as shown in Fig. 9A. The corresponding linear regression equation is Y = 0.05797X + 0.0021, (R^2^ = 0.996, n = 5), linear range is 0.3–16 μM and the detection limit is 0.29 μM. In Table 2, the comparison is made with different electrochemical methods. In order to study the anti-interference of this method, other metal ions and antioxidants possibly existed in white wine, including Ca^2+^, Mg^2+^, Zn^2+^, Cu^2+^, Mn^2+^, Na^+^, K^+^, glucose, fructose, citric acid, tannic acid, 4-methylpyrocatechol and cyanidin cation, were examined. The results were shown in Fig. 9B. In most cases, the interfering substance did not change the absorbance intensity of the system significantly. In addition, we also compared the analytical performance of this method for the detection of *trans*-resveratrol with results in the literature^27,28^. The detection sensitivity of this method is higher than previously reported. The repeatability of the proposed method was evaluated by three replicate measurements of 5 μM of *trans*-resveratrol, and the relative standard deviation (RSD) was 5%, demonstrating the reliability of the proposed method.

**Table 2:** The comparison of different probes for trans-resveratrol detection.

Method	Linear range (μM)	Detect limit (μM)	Reference
Pt electrode	15–120	5.5	^30^
GC electrode	5–75	2.3	^30^
nano-PrO1.8	0.3–16	0.29	This work

### Detection of white wine samples

The content of *trans*-resveratrol in white wine and champagne was determined by the TMB-H_2_O_2_-nano-PrO_1.8_ system. The collected wine was treated with ultrasonic for 10 minutes to remove excess bubbles, and then centrifuged for 5 minutes at 4000 revolutions per minute to remove impurities. The 1 mg/mL *trans*-resveratrol solution was prepared and added into TMB-H_2_O_2_-nano-PrO_1.8_ detection system under optimal reaction conditions. In order to evaluate the accuracy of this method, *trans*-resveratrol solutions with various concentrations were added into diluted white wines samples, and recoveries were calculated. Acetic acid buffer solution was used as a blank sample instead of *trans*-resveratrol solution. Table 3 shows the recoveries of *trans*-resveratrol detected by the TMB-H_2_O_2_-PrO_1.8_ system in the actual sample. The recoveries are between 101.02% and 103.01%, and the RSD is 2.3~5.76%, which indicates the reliability of this method.

**Table 3:** Detection of *trans*-resveratrol in white wine and champagne samples.

Sample	*Trans*-resveratrol spiked (μM)	*Trans*-resveratrol measured (μM)	Recovery (%)	RSD (%, n = 3)
white wine	7.3	7.52	103.01	5.76
	11.68	11.85	101.45	5.35
	14.6	14.85	101.71	2.30
champagne	7.3	7.382	101.12	5.36
	11.68	11.8	101.02	5.20
	14.6	14.82	101.51	2.40

## Conclusions

In this study, the nano-PrO_1.8_ were prepared successfully by ultrasonic as the power, ILs as template and hydrazine hydrate as precipitating agent. The prepared nano-PrO_1.8_ possess high peroxidase-like activity and oxidase-like activity, and the peroxidase-like K_m_ values of the nano-PrO_1.8_ with TMB and H_2_O_2_ were smaller than that of HRP, indicating that the nano-PrO_1.8_ possess better affinity for TMB and H_2_O_2_ than HRP. In addition, *trans*-resveratrol can inhibit the peroxidase-like activity of nano-PrO_1.8_ significantly. On this basis, a reliable and low-cost colorimetric quantitative detection method for *trans*-resveratrol was proposed. The linear range was from 0.3 to 16 μM with a detection limit of 0.29 μM. This work provides a novel method for rapid preparation of peroxidase-like nano-PrO_1.8_ and extends the sensing application of *trans*-resveratrol to the fields of wine samples.
